# p53 protein expression patterns associated with *TP53* mutations in breast carcinoma

**DOI:** 10.1007/s10549-024-07357-z

**Published:** 2024-06-20

**Authors:** Sarah A. Anderson, Brooke B. Bartow, Shuko Harada, Gene P. Siegal, Shi Wei, Valeria L. Dal Zotto, Xiao Huang

**Affiliations:** Department of Pathology, The University of Alabama at Birmingham, 619 19th Street South, Birmingham, AL 35294 USA

**Keywords:** p53 immunohistochemistry, *TP53* mutation, Breast carcinoma

## Abstract

**Purpose:**

The importance of a *TP53* mutation has been demonstrated in several tumor types, including breast cancer (BC). However, the accuracy of p53 protein expression as a predictor of gene mutation has not been well studied in BC. Therefore, we evaluated p53 protein expression associated with *TP53* mutations in breast cancers from 64 patients.

**Methods:**

*TP53* mutation was examined using next-generation sequencing (NGS). p53 protein expression was examined using immunohistochemistry (IHC).

**Results:**

Among the 64 BCs, 55% demonstrated abnormal expression patterns including 27% overexpression, 22% null, 6% equivocal with 45% having a wild-type pattern. A *TP53* mutation was present in 53% (34/64) of tumors including 30% (19/64) demonstrating a missense mutation, 11% (7/64) with a frameshift mutation, 11% (7/64) with a nonsense mutation, and 3% (1/64) with a splice site mutation. Abnormal expression of p53 protein was present in 33 of 34 (97%) tumors carrying a *TP53* mutation; conversely, a wild-type pattern was present in 28 of 30 (93%) tumors without a detectable mutation (*p* < 0.0001). The majority of BCs with a p53 IHC overexpression pattern (15/17, 88%) contained a missense *TP53* mutation; while the majority of BCs with a null pattern (12/14, 86%) contained a truncating mutation (*p* < 0.0001). The BCs with a null pattern are associated with a high Nottingham histological grade and a triple-negative phenotype when compared to those demonstrating overexpression (*p* < 0.05).

**Conclusion:**

These findings suggest that p53 IHC can be a potential surrogate for *TP53* mutations in BC. Different p53 expression patterns may correlate with specific *TP53* genetic mutations in BC.

## Introduction

*TP53* is the most frequently mutated gene identified in most cancer types [1]. p53 protein is a regulatory protein referred as “the guardian of the genome,” involved in multiple physiologic activities including modulating the cell cycle, apoptosis, and genomic stability [2]. Mutations in the *TP53* gene affect tumor suppressor function and confer the oncogenic properties of p53 protein [2]. Approximately 30% of breast cancers (BC) harbor a *TP53* mutation [3]. Studies report that a *TP53* mutation is an independent prognostic marker predicting poor prognosis in BC and to a lesser extent in p53 protein expression [4, 5].

From a practical standpoint, the clinical applicability of p53 expression for patients with BC has yet to be confirmed. The correlation between p53 expression and *TP53* mutation is currently suboptimal [6, 7]. Besides the biological instability of p53 protein with variable detecting assays, the lack of consensus on interpretation is a major issue. The majority of clinical laboratories utilize immunohistochemistry (IHC) to examine the p53 protein expression in breast tumor specimens. The assessment of p53 IHC staining is predominantly based on protein overexpression, and reported as “positive” or “negative,” or provided as a “percentage.” However, accumulating studies have shown that other IHC patterns, such as a complete absence or cytoplasmic expression, can also indicate a *TP53* mutation in several cancer types [7–9]. Optimizing the interpretation of p53 IHC staining is crucial for patient management of BC; therefore, we aim to improve the correlation between p53 IHC expression and *TP53* mutation status, with appropriate interpretation.

## Methods

### Invasive breast carcinoma samples

Patients diagnosed with invasive breast carcinoma between 2014 and 2022 at our institution whose tumors underwent next-generation sequencing (NGS) were identified. For these patients, we used samples of biopsy or surgical excision specimens from the primary or metastatic tumor. Patient age, tumor size, pathologic T, N and M stage, and history of neoadjuvant chemotherapy (NACT) were collected from slide review and the patients’ medical record after institutional IRB approval.

### Histology

All stained slides were reviewed independently by two pathologists (XH and SAA) and the pathologic characteristics were affirmed, including histologic grade, histologic type, and predictive marker status. The American Society of Clinical Oncology/College of American Pathologists guideline recommendations [10–12] were used as references to categorized estrogen receptor (ER), progesterone receptor (PR), and human epidermal growth factor receptor 2 (HER2) status as part of the routine pathologic evaluation. Tumors with ER-low positivity (1–10%) were considered as ER-positive in this study.

### Immunohistochemistry for p53 protein

**Fig. 1:**
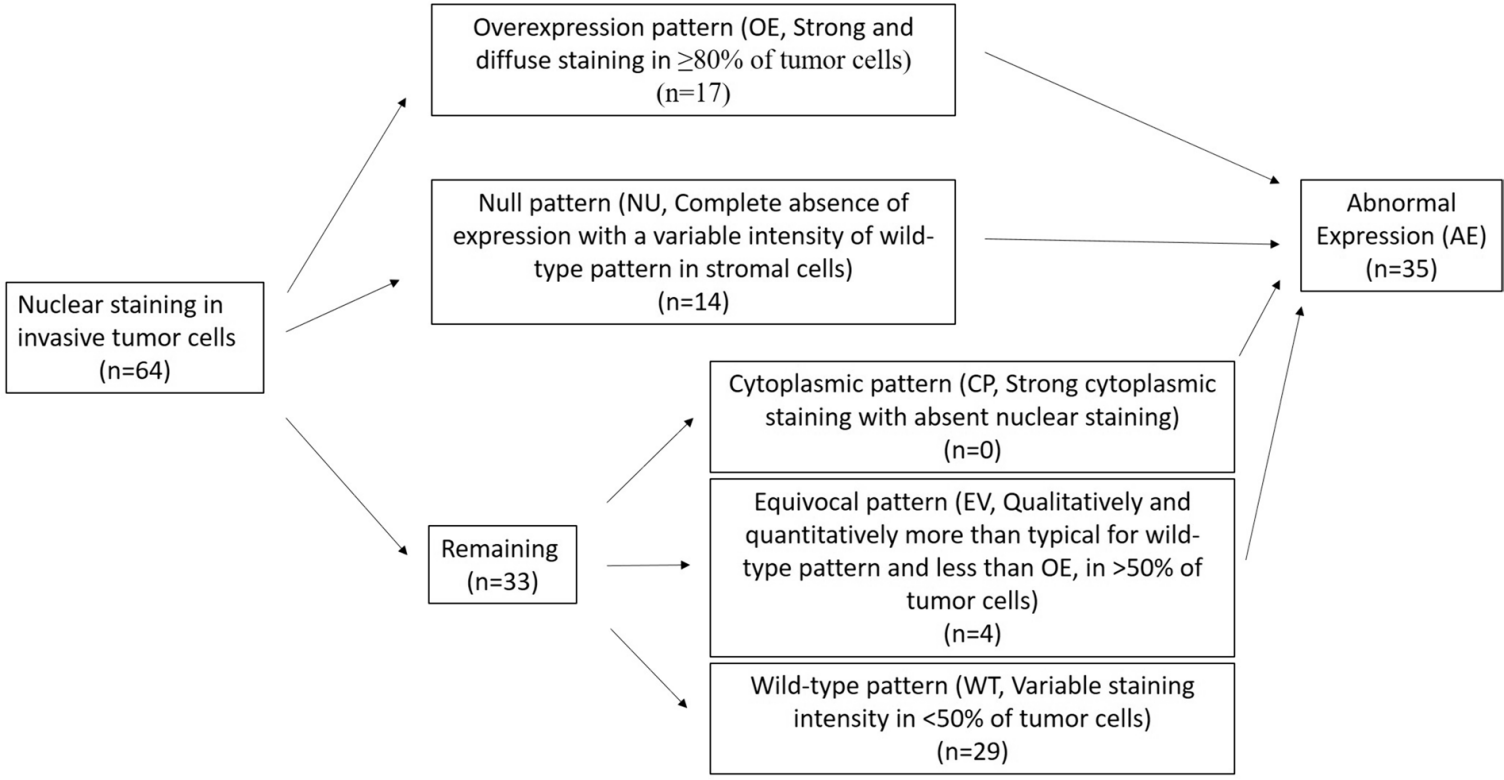
Algorithm for p53 immunohistochemistry interpretation

The p53 IHC was performed on 4 μm thick whole slide sections from formalin-fixed paraffin-embedded (FFPE) tumor tissue using anti-p53 (Bp53-11) antibody (Ventana Medical Systems Inc., Tucson, Arizona). The algorithm for p53 IHC interpretation is shown in Fig. 1. A normal pattern of p53 expression (WT, wild-type pattern) was defined as variable staining intensity in < 50% of invasive tumor cells. Abnormal expression of p53 protein in invasive tumor cells were defined as an overexpression pattern (OE, strong and diffuse nuclear staining in at least 80% of tumor cells), null-type pattern (NT, complete absence of expression, with a variable intensity of staining in stromal cells acting as an internal control) [8, 13], a cytoplasmic pattern (CY, strong cytoplasmic staining with absent nuclear staining), or an equivocal pattern (EV, qualitatively and quantitatively greater than that seen in a wild-type pattern but less than that seen in OE) [7]. For discordant cases, the slides were reviewed independently by 3 pathologists (XH, SAA and VLD).

### Next-generation sequencing for TP53 mutation

NGS analysis was carried out and reported by one of four CLIA-certified laboratories at the request of the treating physicians. FFPE tumors from 28 patients were subjected to whole-exome sequencing (DNA) for detection of single nucleotide variants (SNVs), insertion and deletion alterations (indels), copy number alterations (CNAs), karyotyping, viruses, and whole transcriptome sequencing (RNA) for gene fusions and variant transcripts (Caris Life Sciences, Inc. Phoenix, AZ). FFPE tumors from 23 patients were subjected to DNA extraction and target enrichment for NGS using the Agilent HaloPlex HS targeted sequencing method (Agilent Technologies, Santa Clara, CA). NGS was also performed using the Illumina MiSeq instrument (Illumina, San Diego, CA [14]). Alterations in 50 genes that are relevant for breast cancer were reported (UAB pathology laboratory). FFPE tumors from 8 patients were subjected to a qualitative NGS that uses targeted high throughput hybridization-based capture technology for detection of substitutions, indels, and CNAs in 324 key cancer-related genes, when using the DNAx extraction method (Foundation Medicine Inc., Cambridge, MA). FFPE tumors from 5 patients were subjected to a NGS targeting 323 genes in DNA coding regions. The genomic alterations include SNPs, indels, rearrangements and other alterations (NeoGenomics Laboratories, Inc. Aliso Viejo, CA). All NGS cover the entire exon with at least a 10 base flanking region of *TP53.* In this study, pathogenic and likely pathogenic alterations of *TP53* gene were considered as carrying a pathogenic mutation; gene alterations with a variant of uncertain significance (VUS) were excluded. For data analysis, *TP53* mutation status was subclassified using two-tiers and three-tiers. The two-tier classifier was defined as: *TP53* mutated (any detected pathogenic alteration) or *TP53* wild-type (no detectable mutation). The three-tier classifier was defined as: missense; truncating including frameshift, nonsense and splice site; or no detectable mutation.

### Statistical analysis

A Kruskal–Wallis test was used for group comparisons. A Fisher’s exact test was used to explore the association between two categorical variables. All the tests were two-tailed at a significance level of 0.05.

## Results

We identified a total of 97 patients in our database whose tumors underwent NGS per clinical request. Sixty-four patients had available tumor specimens, on which we performed p53 IHC. IHC and sequencing analysis were independently generated. The interpretation of gene mutations and staining patterns was blinded to the independent molecular pathologists and breast pathologists. The detailed *TP53* mutations and p53 IHC patterns are shown in Table 1.

**Table 1:** Details of *TP53* mutations and p53 IHC patterns

*TP53* mutation type	*TP53* mutation	p53 IHC pattern
Frameshift	p.A159fs	Null
Frameshift	P.C124fs	Null
Frameshift	P152Afs*14	Null
Frameshift	P152Afs*14	Null
Frameshift	P152Afs*14	Overexpression
Frameshift	R213*, S362fs	Overexpression
Frameshift	S95Cfs*	Null
Missense	C176R	Overexpression
Missense	L257P	Overexpression
Missense	M246V	Wild-type
Missense	p.G245D, pY234N	Equivocal
Missense	p.KI32R	Overexpression
Missense	p.P278S	Overexpression
Missense	p.p77r	Overexpression
Missense	p.R156P	Overexpression
Missense	p.R248Q	Overexpression
Missense	p.R273H	Equivocal
Missense	p.T275F	Overexpression
Missense	R175H	Overexpression
Missense	R273H	Overexpression
Missense	R280G	Overexpression
Missense	R280T	Overexpression
Missense	R282W	Null
Missense	V157F	Overexpression
Missense	V216M	Overexpression
Missense	Y220C	Overexpression
Nonsense	E198*	Null
Nonsense	p.E28/*	Null
Nonsense	p.Q144*	Null
Nonsense	p.r213*	Null
Nonsense	p.R342*	Equivocal
Nonsense	p.Y10/*	Null
Nonsense	R213*	Null
Splice site	splice site 560-1G > A	Null

The symbol * represent a type of molecular change

### Clinicopathological features

The clinicopathological features of the patients stratified by p53 IHC expression and *TP53* mutation status (two-tier classification) are shown in Table 2. Of the 64 cases, 35 (55%) cases showed an abnormal p53 IHC expression, and a *TP53* mutation was detected in 34 (53%) of these cases. Nottingham histological grade, ER status, and triple-negative breast cancer (TNBC) were significantly associated with an abnormal p53 IHC expression pattern and *TP53* mutation. Other analyzed characteristics failed to show significant correlations. The comparison of clinicopathological features between cases with a *TP53* missense mutation and those with a truncating mutation are shown in Table 3. A truncating mutation is positively associated with PR negativity and TNBC (*p* = 0.0366, 0.0135, respectively).

**Table 2:** Clinicopathological features stratified by p53 IHC expression and *TP53* mutation status

Factors		p53 IHC		*TP53* mutation status	
		Abnormal Expression	Wild-type pattern	*TP53* mutated	No detectable mutation
Age (mean, range)		58 (30–79)	59 (32–86)	59 (30–86)	58 (32–84)
				*p* = 0.920	
(y)*p* T stage (*N* = 57)	1	8 (14%)	9 (15.8%)	7 (12.3%)	10 (17.5%)
	2	13 (22.8%)	8 (14%)	13 (22.8%)	8 (14%)
	3	3 (5.3%)	6 (10.5%)	4 (7%)	5 (8.8%)
	4	8 (14%)	2 (3.6%)	8 (14%)	2 (3.6%)
		*p* = 0.177		*p* = 0.220	
(y)*p* N stage (*N* = 56)	0	10 (17.8%)	12 (21.4%)	10 (17.8%)	12 (21.4%)
	1	10 (17.8%)	9 (16.1%)	9 (16.1%)	10 (17.8%)
	2	5 (9%)	3 (5.3%)	6 (10.7%)	2 (3.6%)
	3	5 (9%)	2 (3.6%)	5 (9%)	2 (3.6%)
		*p* = 0.672		*p* = 0.398	
(y)p M stage (*N* = 57)	0	26 (45.6%)	23 (40.3%)	26 (45.6%)	23 (40.3%)
	1	5 (8.8%)	3 (5.3%)	5 (8.8%)	3 (5.3%)
		*p* = 0.715		*p* = 0.715	
Histological subtype (*N* = 64)	Ductal	33 (51.6%)	26 (40.6%)	32 (50%)	27 (42.2%)
	Lobular	2 (3.1%)	3 (4.7%)	2 (3.1%)	3 (4.7%)
		*p* = 0.652		*p* = 0.659	
Nottingham histological grade (*N* = 64)	1	0 (0%)	2 (3.1%)	0 (0%)	2 (3.1%)
	2	9 (14.1%)	16 (25%)	9 (14.1%)	16 (25%)
	3	26 (40.6%)	11 (17.2%)	25 (39%)	12 (18.8%)
		***p* = 0.004**		***p* = 0.009**	
ER (*N* = 64)	Positive	16 (25%)	26 (40.6%)	17 (26.6%)	25 (39%)
	Low positive	1 (1.6%)	0 (0%)	1 (1.6%)	0 (0%)
	Negative	18 (28.1%)	3 (4.7%)	16 (25%)	5 (7.8%)
		***p* = 0.0003**		***p* = 0.007**	
PR (*N* = 64)	Positive	16 (25%)	19 (29.7%)	17 (26.6%)	18 (28.1%)
	Negative	19 (29.7%)	10 (15.6%)	17 (26.6%)	12 (18.8%)
		*p* = 0.136		*p* = 0.460	
HER2 (*N* = 64)	Positive	4 (6.2%)	3 (4.7%)	4 (6.2%)	3 (4.7%)
	Negative	30 (46.9%)	27 (42.2%)	30 (46.9%)	27 (42.2%)
		*p* = 1		*p* = 1	
TNBC (*N* = 64)	Yes	16 (25%)	2 (3.1%)	14 (21.9%)	4 (6.2%)
	No	21 (32.9%)	25 (39%)	20 (31.3%)	26 (40.6%)
		***p* = 0.0007**		***p* = 0.02**	

Bold indicates statistically significant

**Table 3:** Comparison of clinicopathological features between *TP53* missense mutation and truncating mutation

Factors		Missense	Truncating
(y)*p* T stage (*N* = 32)	1	4 (12.5%)	3 (9.5%)
	2	8 (25%)	5 (15.6%)
	3	2 (6.2%)	2 (6.2%)
	4	4 (12.5%)	4 (12.5%)
		*p* = 0.957	
(y)*p* N stage (*N* = 30)	0	6 (20%)	4 (13.3%)
	1	5 (16.7%)	4 (13.3%)
	2	3 (10%)	3 (10%)
	3	4 (13.3%)	1 (3.3%)
		*p* = 0.842	
(y)*p* M stage (*N* = 31)	0	14 (45.2%)	12 (38.7%)
	1	4 (12.9%)	1 (3.2%)
		*p* = 0.424	
Nottingham histological grade (*N* = 34)	1	0 (0%)	0 (0%)
	2	7 (20.6%)	2 (5.9%)
	3	12 (35.3)	13 (38.2%)
		*p* = 0.240	
ER (*N* = 34)	Positive	12 (35.3%)	5 (14.7%)
	Low positive	1 (3%)	0 (0%)
	Negative	6 (17.6%)	10 (29.4%)
		*p* = 0.082	
PR (*N* = 34)	Positive	13 (38.2%)	4 (11.8%)
	Negative	6 (17.6%)	11 (32.4%)
		***p* = 0.0366**	
HER2 (*N* = 34)	Positive	4 (11.8%)	0 (0%)
	Negative	15 (44.1%)	15 (44.1%)
		*p* = 0.113	
TNBC (*N* = 34)	Yes	4 (11.8%)	10 (29.4%)
	No	15 (44.1%)	5 (14.7%)
		***p* = 0.0135**	

Bold indicates statistically significant

### p53 IHC patterns

**Fig. 2:**
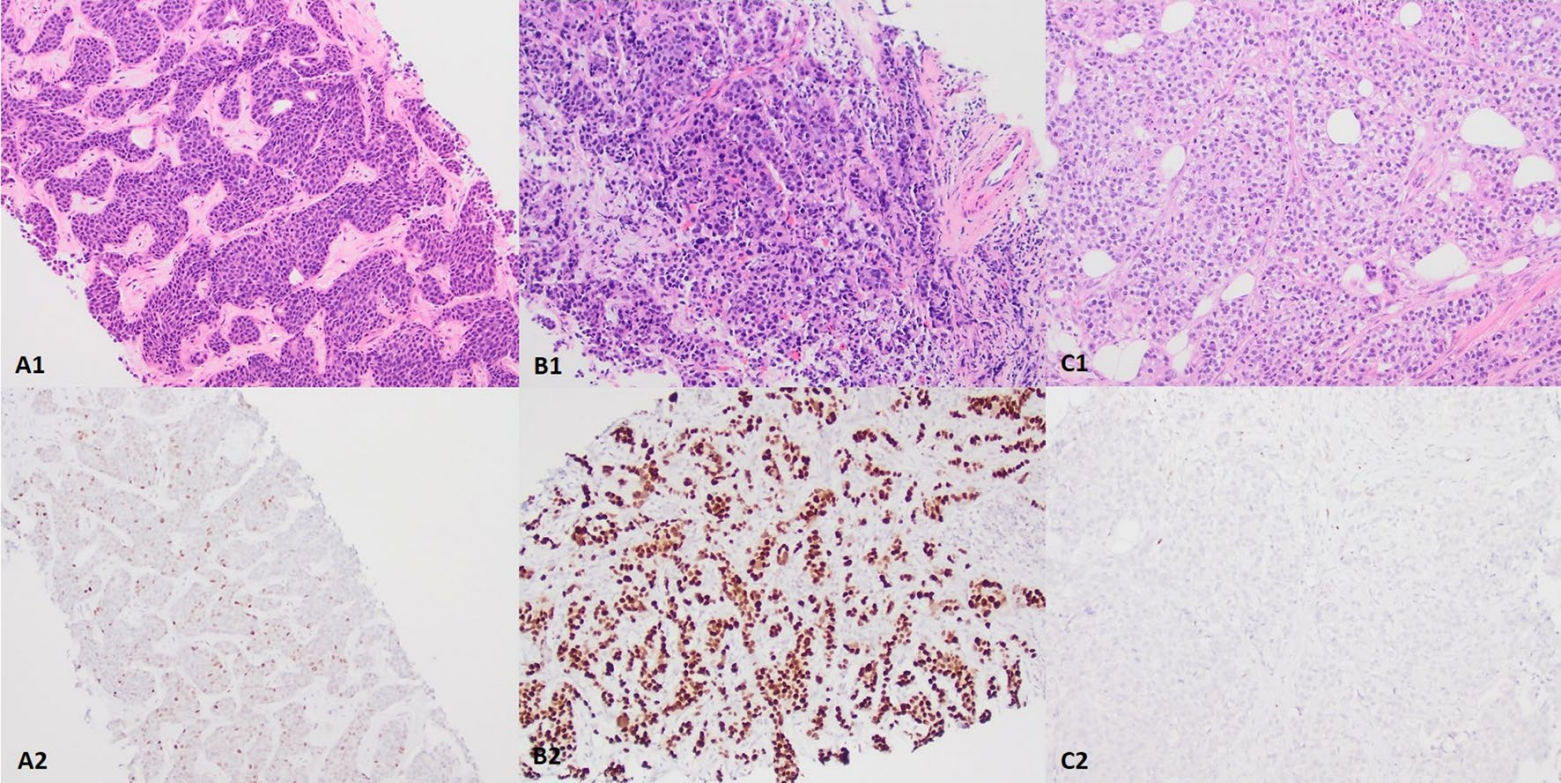
Representative cases showing patterns of p53 immunohistochemical staining. **A** wild-type pattern. **B** overexpression pattern. **C** null pattern. A1-C1, H&E staining (× 100). A2-C2, p53 IHC staining (× 100)

Among the entire cohort of 64 tumors with p53 IHC reactivity, 27% demonstrated an overexpression pattern (17), 22% a null pattern (14), 6% an equivocal pattern (4), and 45% a wild-type pattern (29). All the 17 tumors with a p53 overexpression pattern exhibited unequivocally strong and diffuse nuclear staining in > 80% of the tumor cells. All the 29 tumors classified as p53 wild-type pattern exhibited weak to moderate nuclear staining in < 50% of the tumor cells. There were four tumors which exhibited moderate to strong nuclear staining in > 50% of the tumor cells but did not meet 80% threshold for overexpression. Thus, these four tumors were classified as having an equivocal pattern. None of the tumors exhibited a cytoplasmic pattern. Examples are shown in Fig. 2. The comparison of clinicopathological features between p53 IHC overexpression and null patterns is shown in Table 4. The null pattern is positively associated with TNBC and Nottingham histological grade 3 (*p* = 0.032, 0.045, respectively).

**Table 4:** Comparison of clinicopathological features between p53 IHC overexpression pattern and null pattern

Factors		Overexpression	Null
(y)P T stage (*N* = 29)	1	4 (13.8%)	4 (13.8%)
	2	8 (27.6%)	3 (10.3%)
	3	0 (0%)	3 (10.3%)
	4	4 (13.8%)	3 (10.3%)
		*p* = 0.183	
(y)p N stage (*N* = 27)	0	7 (26%)	3 (11.1%)
	1	4 (14.8%)	4 (14.8%)
	2	3 (11.1%)	2 (7.4%)
	3	2 (7.4%)	2 (7.4%)
		*p* = 0.857	
(y)p M stage (*N* = 28)	0	13 (46.4%)	11 (39.3%)
	1	3 (10.7%)	1 (3.6%)
		*p* = 0.613	
Nottingham histological grade (*N* = 31)	1	0 (0%)	0 (0%)
	2	7 (22.6%)	1 (3.3%)
	3	10 (32.2%)	13 (41.9%)
		***p* = 0.0454**	
ER (*N* = 31)	Positive	10 (32.2%)	4 (13%)
	Low positive	0 (0%)	0 (0%)
	Negative	7 (22.6%)	10 (32.2%)
		*P* = 0.149	
PR (*N* = 31)	Positive	10 (32.2%)	4 (13%)
	Negative	7 (22.6%)	10 (32.2%)
		*p* = 0.149	
HER2 (*N* = 31)	Positive	2 (6.4%)	0 (0%)
	Negative	15 (48.4%)	14 (45.2%)
		*p* = 0.4882	
TNBC (*N* = 31)	Yes	5 (16.1%)	10 (32.2%)
	No	12 (38.7%)	4 (13%)
		***p* = 0.032**	

Bold indicates statistically significant

### Association between p53 IHC patterns and TP53 mutation status

Among the 34 tumors with *TP53* mutations, the overall subclassifications were 56% missense (19), 21% frameshift (7), 21% nonsense (7), and 3% splice site (1). The comparison of p53 IHC patterns with *TP53* mutation status is shown in Table 5A. The concordance was analyzed using a three-tier classification where *TP53* missense, truncating or no detectable mutation (NDM) was compared to overexpression, null-type, equivocal, and wild-type pattern. The prevalence of the each of the specific IHC patterns was different across the three *TP53* mutation subgroups (Table 5B). The p53 overexpression pattern was present in 79% (15/19) of tumors with a missense mutation, 13% (2/15) with a truncating mutation, and 0% (0/30) with NDM (*p* < 0.0001). The p53 null-type pattern was present in 5% (1/19) of tumors with a missense mutation, 80% (12/15) with a truncating mutation, and 3% (1/30) with NDM (*p* < 0.0001). Furthermore, the concordance was analyzed using a two-tier classification schema where the presence or absence of a pathogenic *TP53* mutation was compared to a wild-type pattern or abnormal expression. Abnormal expression was present in 97% (33/34) of tumors carrying a *TP53* mutation (*p* < 0.0001, Table 6).

**Table 5:** Comparison of p53 immunohistochemistry (IHC) patterns with TP53 mutation status

IHC patterns	5A. Overall *TP53* mutation status					5B*. TP53* mutation status (Three-tier classifier)			Total
	Missense	Frameshift	Nonsense	Splice site	No detectable mutation	Missense	Truncating^a^	NDM	
Overexpression	15	2	0	0	0	15	2	0	17
Null	1	5	6	1	1	1	12	1	14
Equivocal	2	0	1	0	1	2	1	1	4
Wild-type	1	0	0	0	28	1	0	28	29
Total	19	7	7	1	30	19	15	30	64

^a^Frameshift, nonsense and splice site mutations are considered as truncating mutation

**Table 6:** Comparison of p53 IHC pattern and *TP53* mutation status using two-tier classification

Two-tier classification		*TP53* mutation status		
		Presence	Absence	Total
p53 IHC	Abnormal expression	33	2	35
	Wild-type	1	28	29
	Total	34	30	64

### p53 IHC equivocal cases and p53 IHC-TP53 mutation status discordant cases

**Fig. 3:**
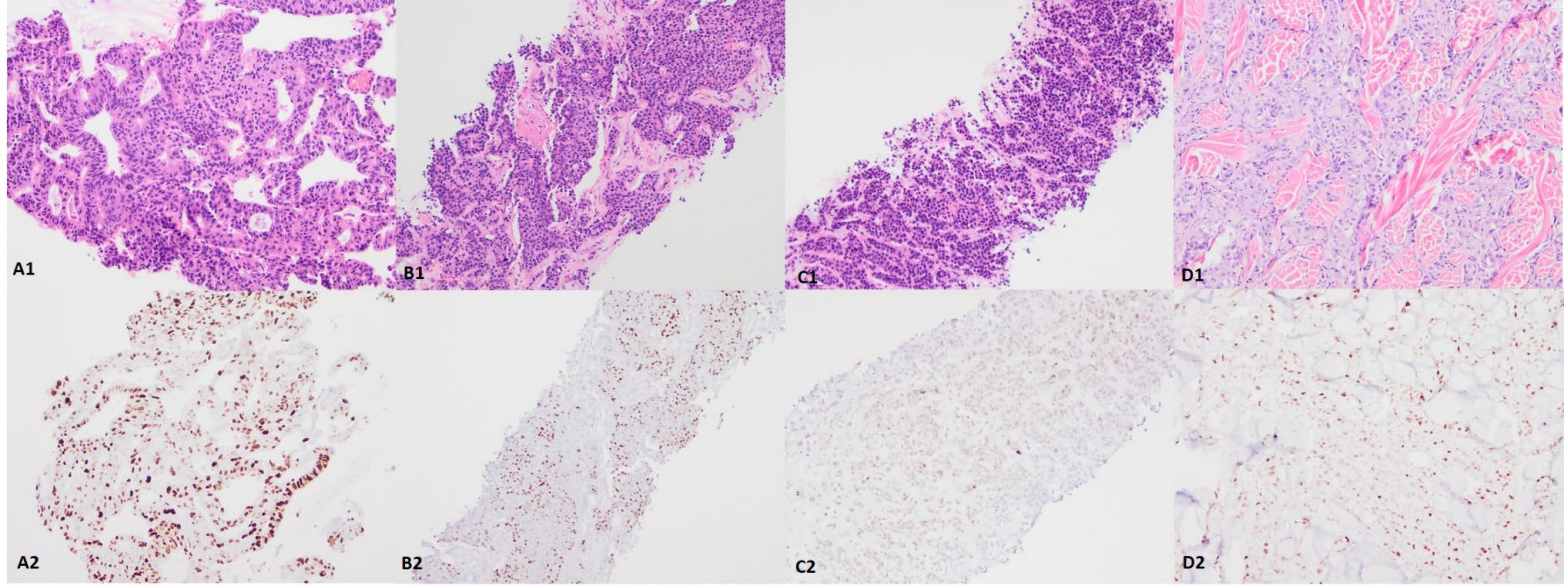
Tumors with a p53 IHC equivocal pattern. A1-A2, Case #1, p53 IHC exhibits nuclear staining with moderate to strong intensity in > 50% but < 80% tumor cells. A *TP53* p.R273H mutation was detected in the tumor. B1-B2, Case #2, p53 IHC exhibits nuclear staining with moderate to strong intensity in > 50% but < 80% tumor cells. A *TP53* p.G245D, p.Y234N mutation was detected. C1-C2, Case #3, p53 IHC exhibits nuclear staining with weak intensity in > 50% but < 80% tumor cells. A *TP53* p.R342* mutation was detected in the tumor. D1-D2, Case #4, p53 IHC exhibits nuclear staining with moderate intensity in > 50% but < 80% tumor cells. A *TP53* mutation was not detected in the tumor. A1-D1, H&E staining (× 100). A2-D2, p53 IHC staining (× 100)

**Fig. 4:**
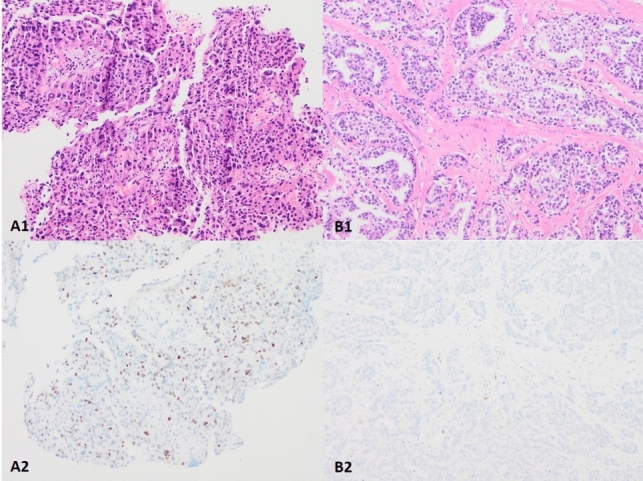
Discordant cases. A1-A2, Case #1, p53 IHC exhibits wild-type pattern as nuclear staining with moderate intensity in < 50% tumor cells. A *TP53* M246V mutation was detected in the tumor. B1-B2, Case #2, p53 IHC exhibits null-type pattern. A *TP53* mutation was failed to detect in the tumor. A1-B1, H&E staining (× 100). A2-B2, p53 IHC staining (× 100)

Four tumors were classified as having an equivocal pattern (Fig. 3). A *TP53* mutation was detected in three of the four tumors. Two tumors had discordant results (Fig. 4). One tumor exhibited a p53 wild-type pattern and a *TP53* M246V mutation was detected. The other tumor exhibited a p53 null-type pattern but a *TP53* mutation was not detected.

## Discussion

This study provides evidence that a p53 IHC assay with appropriate interpretation predicts a *TP53* mutation in breast carcinoma, and that a specific p53 IHC pattern can predict a certain type of *TP53* mutation. Both abnormal p53 IHC expression and a *TP53* mutation show positive correlations with unfavorable pathologic features.

*TP53* mutations are associated with the basal-like subtype and poor overall survival in BC patients [15, 16]. In our study, 34 of the 64 (53%) cases carry a *TP53* mutation, 19 of them (56%) are missense mutations. A *TP53* mutation is associated with a high Nottingham histological grade, ER-negativity, and TNBC, in agreement with previous studies.

Although previous studies showed p53 protein overexpression by IHC was also associated with high risk clinicopathologic features [6, 17], the value of p53 IHC as an accurate predictor of *TP53* mutation in BC has not heretofore been fully established [6]. The majority of the *TP53* mutations leading to abnormal p53 protein accumulation can be detected by IHC. Most of the previous correlative studies evaluated p53 IHC overexpression and interpreted the results semi-quantitatively, but the accuracy was suboptimal [6, 7]. It was proposed that tumors showing absence of detectable p53 protein (null pattern) also should be considered as carrying a possible *TP53* mutation [6]. Köbel et al. optimized their p53 IHC assay, which significantly improved sensitivity and specificity in ovarian carcinoma. They defined abnormal p53 IHC as overexpression, completed absence of expression, or cytoplasmic staining [8]. In our study, 14 of 64 (22%) cases showed a p53 IHC null pattern and 13 of these 14 cases (93%) showed a *TP53* mutation (*p* < 0.00001), compared to 1 of 29 (3%) having a p53 wild-type pattern. Our data further supports the contention that complete absence of p53 IHC staining should be considered as an indication of a *TP53* mutation in breast carcinoma.

The role of *TP53* mutation in cancer development is complicated and two main mechanisms have been implemented. One is inactivation of the tumor suppressive activity of wild-type p53 by a “loss of function” mechanism; the other is acquiring oncogenic activity by a “gain of function” mechanism [2, 3, 7]. Most *TP53* mutations occur in the DNA binding domain and are more frequently caused by missense point mutations [3, 18]. Additionally, studies have shown that certain *TP53* mutation types are associated with specific p53 IHC patterns with missense mutations typically resulting in accumulation of p53 protein, while frameshift, nonsense and splice site mutations may result in a null pattern [7]. Alsner et al. reported that the clinical outcome for breast cancer patients is significantly different based on different *TP53* mutation types [6, 19]. Their studies showed that missense mutations involved in DNA or zinc binding were associated with the worst outcome, while null mutations and missense mutations within structural/conserved domains were associated with similar but significantly worse outcomes compared to patients with wild-type *TP53* [6]. However, the correlations between subtypes of p53 IHC patterns and *TP53* mutations need further investigation in BC. In our study, 88% (15/17) of the tumors with a p53 IHC overexpression pattern had a missense mutation in *TP53*. By comparison, 86% (12/24) of the tumors with a p53 IHC null pattern had a truncating mutation (*p* < 0.00001). We also identified that tumors with a truncating mutation are more frequently associated with PR negativity and TNBC, compared to those with a missense mutation. As we expected, those cases with a p53 IHC null pattern was positively associated with TNBC and/or Nottingham histological grade 3 compared to those with an overexpression pattern. Further, we note that a p53 IHC staining pattern can predict a specific type of *TP53* mutation in BC, i.e., a p53 IHC null pattern is more frequently associated with a *TP53* truncating mutation and is associated with unfavorable pathologic features.

Among the 64 cases, 2 tumors showed a discordant p53 IHC pattern/*TP53* status. The first tumor had a missense M246V mutation, the p53 IHC showed a wild-type pattern (Fig. 4A). The *TP53* M246V missense mutation obscured structure stability [20], resulting in reduced protein thermostability and loss of DNA binding ability [2, 21]. The p53 IHC failed to show an overexpression pattern, which we would have expected to see in tumors carrying a missense mutation. One possible explanation for this is mutated p53 protein degradation due to poor or delayed formalin fixation of the tissue [6, 8]. The second tumor was p53 IHC null-type, but the NGS failed to reveal a *TP53* mutation (Fig. 4B). We speculate on 3 possible explanations for this discrepancy. Firstly, this could be false null-type p53 IHC in tumor cells, although the internal stromal cells showed wild-type staining. Secondly, this could be related to the tumor cellularity effected by the admixed stromal cells and lymphocytes in the specimen subjected to NGS (false negative of NGS). Lastly, this could be related to our sequencing approach, which may not have been able to detect all the mutations [22]. The NGS approach may not be able to detect loss of an entire gene or its chromosomal location.

Among the 64 cases, 4 tumors showed equivocal p53 IHC staining (Fig. 3). Three of these 4 tumors had detected *TP53* mutations. A missense *TP53* mutation in p.R273H was detected in cases #1 (Fig. 3A) and missense mutations in p.G245D and p.Y234N were identified in case #2 (Fig. 3B). Both of the cases showed increased p53 IHC nuclear staining more than typically seen with a wild-type pattern (moderate to strong intensity in > 50% but < 80% of the tumor cells), but failed to reach the threshold identified with an overexpression pattern. Again, the possible explanation for this is mutated p53 protein degradation due to poor or delayed formalin fixation of the tissue [6, 8], as previously mentioned. Elsewhere, a nonsense *TP53* p.R342* mutation was detected in case #3 (Fig. 3C), the p53 IHC showed weak nuclear staining in > 50% of the tumor cells. This tumor carried a nonsense *TP53* mutation with increased p53 IHC. We speculate a possible explanation being the abnormal p53 protein truncated at the tetramerisation domain within the C-terminus (p.R342*) which may be detected by the p53 Bp53-11 antibody (which binds the transcription domain within the Nh2-terminus). Thus, the IHC staining failed to show a null-type pattern. Lastly, case #4 (Fig. 3D) showed moderate intensity in > 50% but < 80% of the tumor cells by p53 IHC staining with no detectable *TP53* mutation. This increased p53 IHC staining could be due to overestimation. For cases with equivocal p53 IHC staining, NGS should be utilized, we suggest, to determine the *TP53* mutation status.

In summary, using different IHC patterns to link p53 expression with *TP53* mutation status have been proposed and optimized in gynecological and colorectal cancers [7]. Three main patterns of p53 IHC expression are overexpression, null, and wild-type. More recently, additional two patterns have been described in few studies. Köbel et al. [8] and Rabban et al. [13] reported a cytoplasmic pattern which was defined as “strong cytoplasmic staining without nuclear overexpression” in gynecological cancers. Tessier-Cloutier et al. recognized and defined a “basal overexpression” pattern as “uniformly strong nuclear staining in at least 80% of basal cells without significant parabasal staining” in vulvar squamous cell carcinoma [23]. In this study, we propose four p53 IHC patterns: overexpression, null, equivocal and wild-type. We report the first correlational study of p53 IHC patterns and *TP53* mutation status in the context of pathological features in breast carcinoma.

## Conclusion

This study suggests that a p53 IHC assay, with appropriate interpretation, can predict *TP53* mutation status in a large subset of breast carcinomas. Further, a p53 null pattern is associated with a truncating mutation in the *TP53* gene and signals worse pathologic features, compared to cases with an overexpression pattern. For the cases with an equivocal p53 IHC expression, further validation studies are required. Additionally, future investigations are necessary to evaluate for the potential diagnostic and therapeutic significance of different p53 IHC patterns and different types of *TP53* mutations in breast cancer.

## Data Availability

All data generated or analyzed during this study are included in this article and its online supplementary material. Further inquiries can be directed to the corresponding author.
